# The Physical Basis for pH Sensitivity in Biomolecular Structure and Function, With Application to the Spike Protein of SARS-CoV-2

**DOI:** 10.3389/fmolb.2022.834011

**Published:** 2022-02-18

**Authors:** Jim Warwicker

**Affiliations:** School of Biological Sciences, Faculty of Biology, Medicine and Health, Manchester Institute of Biotechnology, University of Manchester, Manchester, United Kingdom

**Keywords:** pH dependence, protein electrostatics, SARS-CoV-2, spike protein, coronaviruses

## Abstract

Since pH sensitivity has a fundamental role in biology, much effort has been committed to establishing physical models to rationalize and predict pH dependence from molecular structures. Two of the key challenges are to accurately calculate ionizable group solvation and hydration and then to apply this modeling to all conformations relevant to the process in question. Explicit solvent methods coupled to molecular dynamics simulation are increasingly complementing lower resolution implicit solvent techniques, but equally, the scale of biological data acquisition leaves a role for high-throughput modeling. Additionally, determination of ranges of structures for a system allows sampling of key stages in solvation. In a review of the area, it is emphasized that pH sensors in biology beyond the most obvious candidate (histidine side chain, with an unshifted p*K*_*a*_ near neutral pH) should be considered; that modeling can benefit from other concepts in bioinformatics, in particular modulation of interactions and function in families of homologs; and that it can also be beneficial to incorporate as many experimental structures as possible, to mitigate against small variations in conformation and to analyze larger, functional, conformational changes. These aspects are then demonstrated with new work on the spike protein of SARS-CoV-2, looking at the pH dependence of variants, including prediction of a change in the balance of locked, closed, and open forms at neutral pH for the Omicron variant spike protein.

## Introduction

Examples of pH dependence occur throughout biological systems. The narrow control of key properties, such as blood pH, mediated by various sensors, transporters, and channels (Rajkumar and Pluznick, 2018), or cytosolic pH (Chiche et al., 2010) points to the importance of pH in underlying molecular processes. Gradients of pH at a cell’s outer membrane or within mitochondria underlie some of the most basic processes of metabolic energy transduction, mechanistically with structures demonstrating a probable proton pathway for mitochondrial ATP synthase (Klusch et al., 2017). Further, more complex cell types display an array of membrane-enclosed subcellular organelles that compartmentalize sets of macromolecules and small molecules, including protons and pH (Demaurex, 2002). Global properties of organelle sub-proteomes, including pI distributions (Kurotani et al., 2019) and predicted maximal pH-dependent stability (Chan and Warwicker, 2009) correlate with their environmental pH. Differences in acidification of the import and export pathways and organelles of eukaryotes are linked to processes that include altered protease activation and receptor/ligand cycling under pH control (Mellman, 1992).

In some cases, sufficient information is available to allow redesign of systems toward a targeted endpoint. An important example is lengthening the circulation half-life of protein therapeutics (biologics), making use of (and extending) pH-dependent recycling of IgGs and serum albumin by the neonatal Fc receptor (FcRn) (Pyzik et al., 2019). Other efforts are underway to engineer systems for altered performance with regard to pH, often screening for successful designs from high-throughput mutagenesis. The pH dependence of antibody–antigen binding can be altered, for example, with increased binding at the acidic pH of the (extracellular) tumor environment (Sulea et al., 2020), or with design aimed specifically at the generation of a pH switch (Strauch et al., 2014). These types of studies are enhanced by advances in fluorescent protein technology that allow increasingly sensitive analysis of extracellular and intracellular pH values and which themselves benefit from molecular engineering (Bencina, 2013). Enzyme pH dependence can be engineered through directed evolution for adaptation to particular tasks in biotechnology or synthetic biology (Scheiblbrandner et al., 2017). Computational design and experimental validation studies have been combined in a study that adjusts buried histidine environments using the Rosetta suite of design tools to control conformation and oligomerization through pH switching (Boyken et al., 2019). Even this study, though, starts with the rather simple observation that histidine side chains are the most notable candidates for pH sensing (owing to their normal p*K*_*a*_ of 6.3) and that ionization of buried groups leads (in the absence of very precise complementary interactions in the immediate vicinity) to instability.

This work will examine the computational methods used to predict pH dependence in proteins, comparing their maturity with other techniques of structural bioinformatics, and will discuss examples of naturally occurring pH dependence, emphasizing the lessons that can be learned when further developing prediction tools. Then, a system that is currently challenging the scientific community on multiple fronts is studied, SARS-CoV-2 and specifically the spike protein. Emerging evidence of a role for pH switching between conformational forms, potential molecular sources of that pH dependence, and how these might be changing in variants are discussed.

### Computational Methods for Predicting pH Dependence

Buried histidine side chain involvement in various biological systems has been experimentally characterized, including conformational stability and disease susceptibility in prion protein diseases (Malevanets et al., 2017), structural core stabilization in a K-homology module RNA binding motif (Fraternali et al., 1999), pH sensing in the Cnu transcription factor (Narayan and Naganathan, 2018), and probing transmembrane protein helical structure (Afrose et al., 2021). In some studies, groups such as buried histidine side chains are described as electrostatically frustrated (depending on the pH) (Gopi et al., 2018; Narayan et al., 2020), a concept also used in application to viral protein structure (Hebditch and Warwicker, 2020). It seems that burial from solvent of an ionizable group with p*K*_*a*_ close to physiological pH, which is normally around neutral in the cytoplasm, goes a long way in identifying regions of pH sensitivity. Indeed, it may be that there exists more scope for bioinformatics searches for pH sensors purely on this basis, perhaps with extension to incorporate non-buried histidine side chains, as has been suggested for arginine-to-histidine somatic mutations in cancer adaptation to metabolic-induced pH changes (White et al., 2017). This philosophy has been successfully employed to study several systems, including pH sensors in Ras-specific guanine nucleotide exchange factor (Vercoulen et al., 2017) and in *β*-catenin (White et al., 2018).

All aspects of sequence and structural bioinformatics are now informed by the plethora of data available, which is why finding a model for searching those data is so valuable. Sequence analysis has benefited enormously from Kyte–Doolittle (hydrophobicity scale) prediction of transmembrane domains (Kyte and Doolittle, 1982) and the incorporation of hydrophobicity and charge for identification of protein regions likely to be intrinsically disordered (Uversky et al., 2000). Prediction of pH dependence requires an estimate of the balance of interactions between ionized and non-ionized forms of pH-titratable groups, in each of the states being considered (Antosiewicz et al., 1994). Typically, these states might be folded and denatured protein (folding), or complex and non-complex (binding), or two different folded conformations. Net electrostatic interactions between ionizable groups are often approximated as zero in the unfolded state, although this can be a problem (Tan et al., 1995). Methods for predicting pH dependence have been developed based on continuum electrostatics approximations for protein and solvent (Warwicker and Watson, 1982). One such implementation (Warwicker, 2004), used in the p*K*_*a*_ application of the protein–sol tool (Hebditch and Warwicker, 2020), incorporates a scheme for partitioning electrostatic interactions (Bashford and Karplus, 1990) and derives p*K*_*a*_s with Monte Carlo sampling of ionization states (Beroza et al., 1991). There are many other implementations of a wide variety of continuum electrostatics (implicit solvent) methods, with reviews available, for example, general (Alexov et al., 2011; Gunner and Baker, 2016) and focused on the generalized Born method (Onufriev and Case, 2019). A major advantage of continuum methods is that their speed allows application in high-throughput studies, aligned to the modern biology of omics data collection. They are complementary to the growing use of lower throughput constant-pH molecular dynamics methods (Chen et al., 2014), which explicitly couple pH titration to conformational change, the central aim of understanding and predicting pH sensing. There is room for both low- and high-throughput methods, not only because of the facilitation of omics scale scans with implicit solvent techniques but also due to the scale of conformational change that may be linked to pH dependence, which is challenging for simulation, but where the experimental structure databases (and perhaps AlphaFold (2) (Jumper et al., 2021) or other modeling schemes) can play a role. In particular (for larger systems), cryo-electron microscopy (cryo-EM) structures are appearing at pace. Work with SARS-CoV-2 spike protein, in a subsequent section, refers to the great variety of information available when structures are solved under multiple conditions, including pH, ligand binding, and mutation.

Beyond the clear relevance of histidine side chains, several classes of ionizable groups need to be considered in models. Certain systems more or less eschew histidine altogether, for example, the thioredoxin and DsbA family of disulfide oxidoreductases, where cysteine p*K*_*a*_ couples to a scale of redox potential and range of biological function that varies substantially through the family (Gane et al., 1995; Collet and Bardwell, 2002). This example highlights the importance of studying models in the context of families and homologs. A common chemistry, disulfide bond making and breaking, and partial stabilization of a cysteine thiolate at the amino terminus of an *α*-helix, is supplemented with sequence and small structural changes within a common fold to modulate p*K*_*a*_ and redox potential. A comparable example is the variation in heme group redox potentials, successfully captured with electrostatics calculations (Zheng and Gunner, 2008). As a side note, cysteine p*K*_*a*_ and reactivity (and that of other groups) appear to be important for the growing field of non-covalent inhibitor development in therapeutics (Fowler et al., 2017).

Aspartic and glutamic acid side chains are obvious candidates for pH sensing, well known from studies of acid–base catalysis in enzyme active sites, often contributing to pH bell curves of activity, perhaps the prototype example being E35 (p*K*_*a*_ 6.3) of lysozyme (Bashford and Karplus, 1990; Bartik et al., 1994). Experimental determination of p*K*_*a*_s in proteins, focusing on NMR, has been reviewed (Hass and Mulder, 2015). Other than catalytic systems, multiple instances of functional ionization for carboxylate side chains have been noted. Amino acids E69 and E74 potentially mediate pH dependence (in the pH range of 4.5–7.1) of coiled-coil formation in a variant of influenza hemagglutinin (Higgins et al., 2014). Association of some antigenic peptides and MHC complex class II proteins is pH-dependent, with buried interfacial Asp/Glu side chain p*K*_*a*_s > 7 thought to be responsible (Belmares et al., 2000). In photosystem I, interfacial residues D612 and E613 of PsaB are proposed to modulate acidic pH-dependence of electron transfer in complexes with plastocyanin or cytochrome *c*_6_ (Kuhlgert et al., 2012). Two Asp side chains in the periplasmic chaperone HdeA of *Escherichia coli* mediate dimer dissociation from neutral to lower pH (Foit et al., 2013). Aspartic acid ionization is coupled to membrane protein function, for example, in G-protein-coupled signal transduction (Dror et al., 2011), and peptide insertion into a membrane (Teixeira et al., 2016) (Vila-Vicosa et al., 2018). Furthermore, protein–protein interactions can be influenced by the Asp charge state (Lin et al., 2017). It is apparent that carboxylate side chains should be considered alongside histidine side chains when considering pH dependence in biology, with the caveat that a degree of burial from solvent will normally be required to elevate a carboxylate p*K*_*a*_ toward neutral pH. In support of this, an engineered, buried, V66E mutation (p*K*_*a*_ 8.8) in staphylococcal nuclease introduces an acidic pH dependence of stability (Dwyer et al., 2000).

Coupling between pH sensors and their environment is a theme that was established early on, with a landmark study of the mutations arising when influenza viruses were selected for their ability to grow in cells with elevated endosomal pH, from treatment with amantadine hydrochloride (Daniels et al., 1985). A large number of the resulting mutations were present at the interface between hemagglutinin subunits and were not themselves ionizable groups. This result makes sense in that the energetics of a process that is susceptible to pH switching (conformational change, complex formation) is coupled to the pH range over which switching occurs. However, it rather complicates the issue of screening specifically for pH-sensing residues through analysis of experimental selection based on altered pH or through large-scale mutagenesis aimed at identifying pH sensors. A similar situation is apparent in acid-sensing ion channels (ASICs), where the results of moderate-scale single-site mutagenesis have been interpreted as reporting on both pH-sensing amino acids (those directly involved as part of a pH sensor) and pH-coupled amino acids (those present in regions that change upon pH switching) (Sazanavets and Warwicker, 2015).

Thus, predictions of pH dependence are more complicated than simply identifying ionizable groups with a normal p*K*_*a*_ value close to physiological pH (typically histidine) and that are buried from solvent. These complicating factors include sufficiently precise calculation of the balance of solvating and desolvating (dehydrating) interactions (be it with implicit or explicit solvent methods), incorporation of enough detail of changes in conformation/complexation (whether using molecular simulation/modeling methods or multiple experimental structures), consideration of the energetic coupling between structural changes and pH sensing (where experimental screens for altered pH dependence will not necessarily distinguish between them), and making sure that models do not exclude classes of groups that may be relevant (for example, carboxylate groups have well-established roles in pH dependence beyond catalytic sites). These areas are also opportunities for further development of prediction methods. A multiplicity of implicit and explicit solvent methods will facilitate implementation of combined high- and low-throughput hierarchical analyses of pH sensing, especially in regard to studies aimed at characterization of pH dependence across proteomes, as more structures and effective models become available. Included in the increasing structural database are ever more snapshots of systems under different conditions, revealing the conformation/complexation changes that pH switches act upon. Experimental screens for altered pH dependence in biological systems, making use of genomics era technologies, although not delivering uniquely pH-sensor groups, should highlight regions that couple to pH-sensing, itself a valuable insight. Supporting all areas is the common theme of bioinformatics, homology, whereby variation within a common framework and evolutionary adaptation, simplifies computation through an analysis of results for closely related systems.

### pH Dependence of SARS-CoV-2 Spike Protein Conformation

Viruses must enter a cell and release their genome for copying. They must also exit the cell. For both entry and exit, pH-dependent processes may play a role, including receptor-mediated endocytosis into low-pH endosomes and navigation of acidic pH in the secretory pathway (Robinson et al., 2018). For the membrane-enveloped coronavirus family, cell entry may be through fusion directly at the cell membrane or through fusion at an intracellular membrane, subsequent to receptor-mediated endocytosis of the virus (Whittaker et al., 2021). Although there is evidence that altering endosomal pH impedes viral entry to some extent (Prabhakara et al., 2021) for the SARS-CoV-2 coronavirus (causative agent of the COVID-19 pandemic), the precise balance of genome release routes (cell surface or interior) may depend on other factors, such as the priming cleavage of S1 and S2 subunits of the spike (S) protein (Papa et al., 2021). For membrane-enveloped viruses, mechanisms evolve to protect against mistimed low pH-induced membrane fusion events in the acidic pH secretory pathways for newly synthesized viruses (Fields and Kielian, 2015). It is apparent that questions of pH dependence in these systems are complex, relate to pH inside and outside of the cell, and are coupled to other determinants of stability, including receptor and other ligand binding and proteolytic cleavages. A strong signal in calculations of pH dependence, relative to the unfolded state, for each of the pre- and post-fusion structures of SARS-CoV-2 S protein are three His side chains in the S2 subunit, but since their burial and predicted pH dependence are uniform between pre- and post-fusion forms, it is unclear whether they are functionally relevant (Warwicker, 2021). For coronaviruses, conformational variation of pre-fusion S protein is apparent from cryo-EM structures. Indeed, there are major questions to be answered about the pH dependence of the S1/S2 subunit S-protein structure and function, even without considering the large-scale conformational changes of the S2 subunit that follow S1 subunit release and accompany membrane fusion (Cai et al., 2020).

Based on the response of the S protein trimer structure to acidic pH (Zhou et al., 2020; Qu et al., 2021), a scheme has been suggested in which the trimers of a virus within the acidic pH of the secretory pathway would be protected in a more tightly packed (locked) conformation (Qu et al., 2021). This suggestion is consistent with the discovery of a locked and more tightly packed form of the SARS-CoV-2 S protein trimer at the normal pH (7.5–8) of structure solution, due to binding of a linoleic acid pocket factor in the receptor binding domain (RBD) (Toelzer et al., 2020). These observations prompt questions about which S protein amino acids are sensing pH, and how these might be coupled to pocket factor binding and packing/interface changes. Such questions highlight areas discussed in this article: regional differences in pH (inside and outside of cells), ligand binding, and interface variation identified with multiple structures and, as will become apparent, the potential influence of mutations (in the S protein). A study of S protein trimers grouped according to RBD down (with locked and closed forms treated separately), and RBD up, reported differences in the predicted pH dependence (Lobo and Warwicker, 2021). The largest contributions to these differences arose from interface tightening in the trimer between closed and locked forms, rather than the well-known difference between open (RBD up) and closed (RBD down) forms, and specifically from Asp and Glu side chains, prominent among which is D614. It was proposed that these carboxylate groups have relatively unperturbed p*K*_*a*_s in open and closed forms, but that increased burial in locked forms leads to destabilization. Depending on the balance of overall interface energetics, the effect could be to favor locked forms at acidic pH (less destabilized carboxylates, pH closer to their normal p*K*_*a*_), and closed/open forms at neutral pH (since locked form carboxylates would be more destabilized, with pH further from their normal p*K*_*a*_) (Lobo and Warwicker, 2021), a similar proposal to that of protection in the secretory pathway (Qu et al., 2021).

**FIGURE 1:**
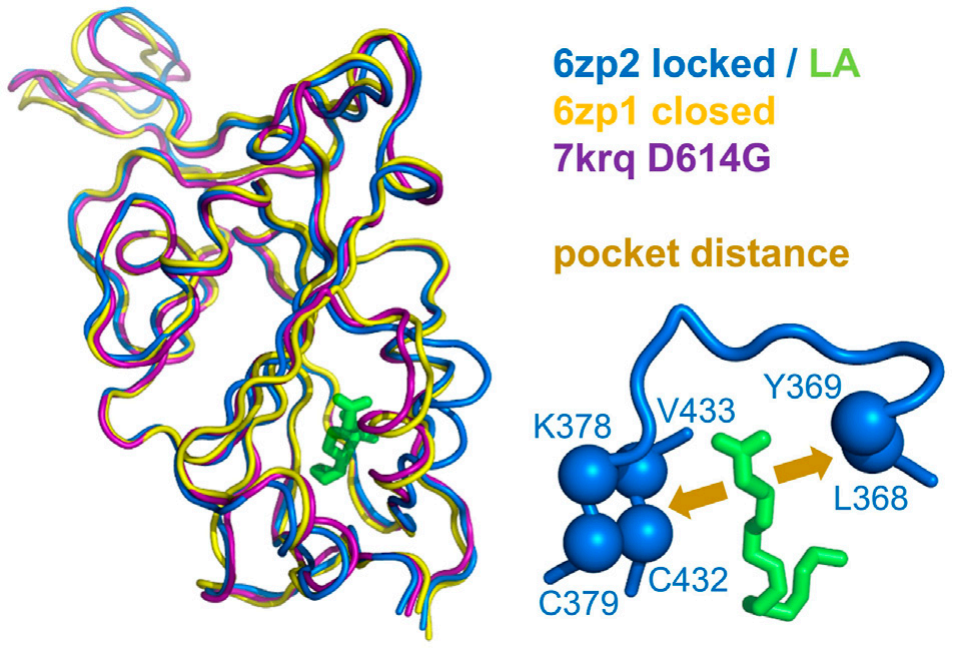
Measure of pocket opening in the RBD. Receptor binding domains are aligned (Swiss PDB Viewer) (Guex and Peitsch, 1997) and drawn (PyMol) from representative locked (6zp2) and closed (6zp1) (Xiong et al., 2020) S protein trimers, and also from an S trimer structure carrying the D614G mutant, 7krq (Zhang et al., 2021a), with all RBDs down and monomer burial approaching the locked form. Distance across the pocket is shown schematically for 6zp2, with linoleic acid (LA) bound. The distance is calculated between the average of the four *β*-strand C_α_ atoms displayed (left of LA, which align well structurally between these RBDs), and the average of the 2 C_α_ atoms shown to the right of LA, present on a turn within a substructure that gates the LA binding pocket (Toelzer et al., 2020).

**FIGURE 2:**
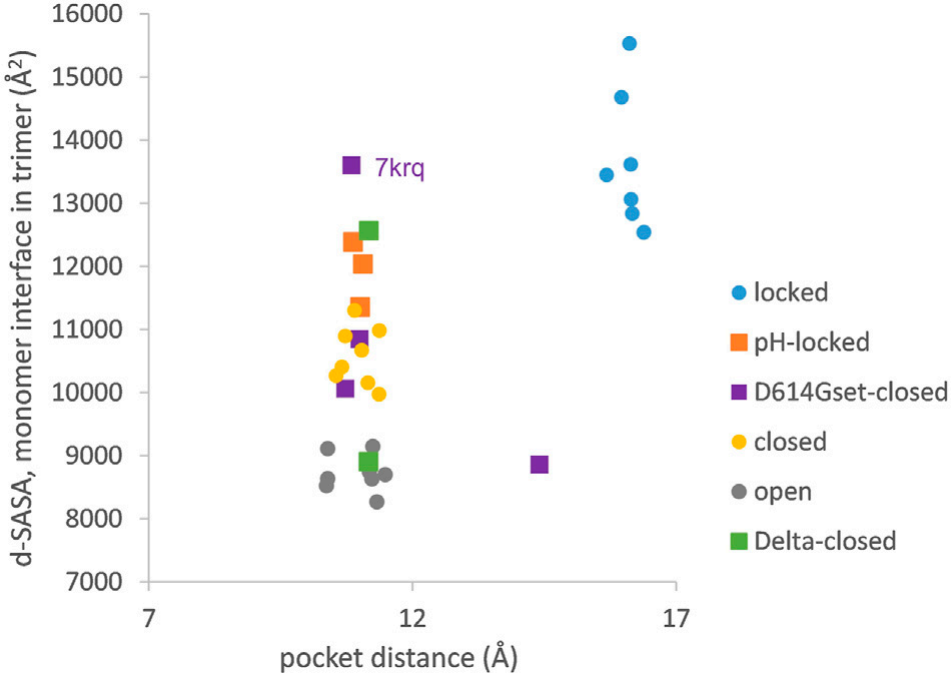
Variants carrying the D614G mutation do not cluster on a plot of pocket distance against S monomer burial within the S trimer. Clustering of locked, pH-locked, closed, and open forms in terms of monomer burial (Lobo and Warwicker, 2021) extends also to pocket distance. However, D614G set S proteins show greater variation, and the two Delta variant S proteins displayed are also well separated in monomer burial. The Delta variant S proteins are additions, while other data points are three monomer averages for each of the S protein trimers in subgroups described previously (Lobo and Warwicker, 2021). Other than the open set, only trimers with all RBDs down are included (and thus sets are named D614Gset-closed and Delta-closed).

The picture of the SARS-CoV-2 spike protein encountered in the first year of the COVID-19 pandemic is therefore of a trimer that can exist in a more tightly packed form (termed locked) either with pocket factor binding at neutral pH or at acidic pH in the absence of pocket factor. However, the spike protein is changing, leading to the question of what the consequences may be for the relative stability of the locked, closed, and open forms, which is the subject of this section. Predictions for potential acid sensor residues are already available (Lobo and Warwicker, 2021). Here, the focus is on how these are situated in the context of interface and RBD pocket changes. Data from previous work for the change in solvent accessible surface area (d-SASA) upon spike monomer incorporation into trimers (in various trimer forms) are supplemented with equivalent calculations for two recent structures of the Delta variant (RCSB/PDB (Berman et al., 2007) identifiers 7v7n (10.2210/pdb7V7N/pdb) and 7sbk (Zhang et al., 2021b)). A new measure is constructed of the distance across the RBD pocket in which linoleic acid can bind (Figure 1). Comparing pocket distance and monomer interface in a scatterplot (Figure 2), it is apparent that open and closed forms cluster with a distance consistent with a closed pocket and at low (open) and intermediate (closed) monomer burial in the trimer. Locked forms are quite separate, with a larger (and occupied) pocket and a high monomer burial. The set of pH-locked forms have closed pockets and monomer burial intermediate between closed and locked forms, but at an acidic pH (all other data in the plot are for structures at or slightly above neutral pH). Structures in both the D614Gset-closed and Delta-closed groups carry the D614G mutation and have been filtered for trimers in which all RBD are down. For both groups, the clustering exhibited by other forms is lost, the monomer burial can vary substantially, and one structure also has an intermediate pocket distance. Variants with the S protein D614G mutation increase recovery in cryo-EM structure determination of forms that approach a locked degree of monomer interface burial but at neutral rather than acidic pH (Lobo and Warwicker, 2021), with evidence that they are also able to sample conformations of intermediate pocket closure (Figure 2). It seems that the barrier between locked and closed forms at neutral pH is reduced by the D614G substitution.

**FIGURE 3:**
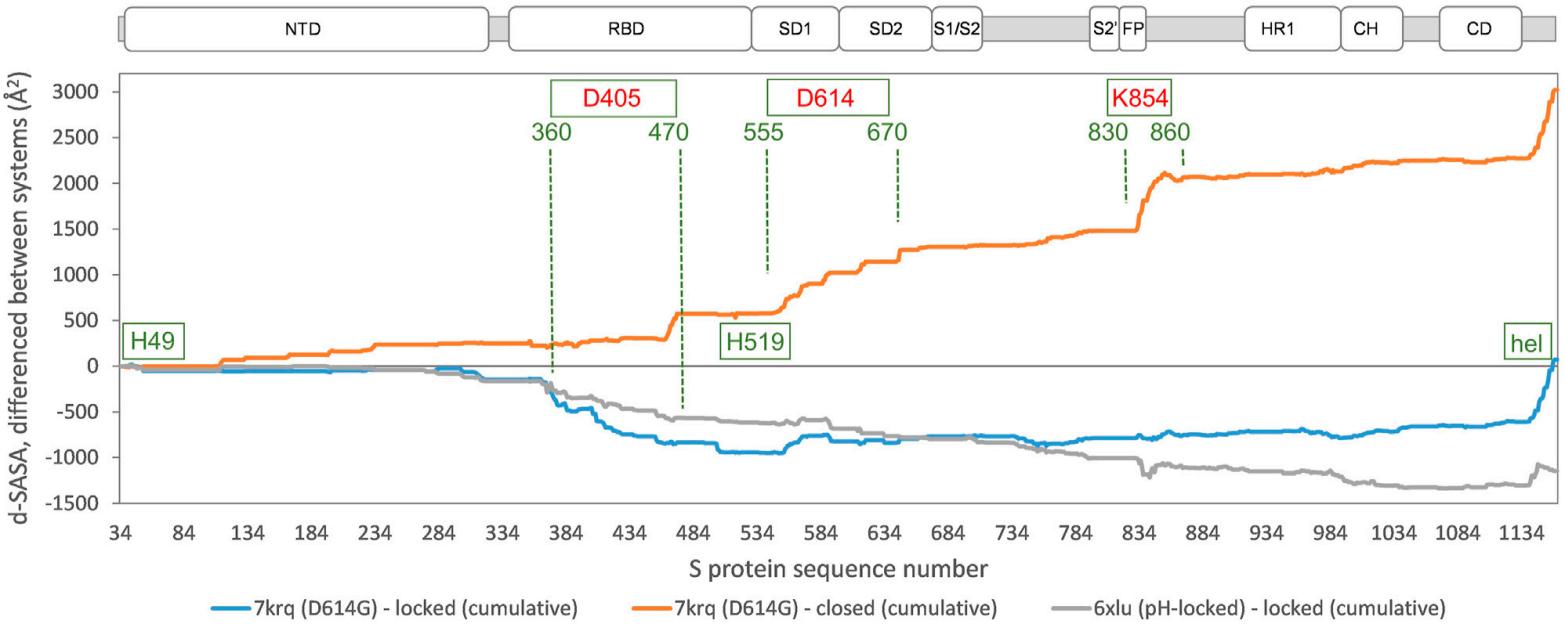
Comparison of acidic pH and D614G mutation effects on packing within the S trimer. The d-SASA value (monomer burial within a trimer) is further differentiated between the systems indicated, between 7krq (D614G, closed pocket but burial approaching the locked form) and the average over the locked set, between 7krq and the average over the closed set, and between 6xlu (pH-locked) and the average over the locked set. These double difference quantities are plotted cumulatively over the sequence of the S trimer, for which the structure is available, with subdomains (Berger and Schaffitzel, 2020) indicated (NTD N-terminal domain, SD1/SD2 subdomains 1 and 2, S1/S2 proteolytic cleavage site between subunits, S2’ cleavage site within S2 subunit, FP fusion peptide, HR1 heptad repeat 1, CH central helix, and CD connector domain). Regions and amino acids of particular interest are displayed with residue numbers and in relation to the changes in burial. A C-terminal helical coil interaction between monomers (present only in some structures) is labeled (hel).

In order to establish the location of interface differences (for monomer burial in trimer) and how they change in a D614G structure with similar burial to locked forms, differences between 7krq (Zhang et al., 2021a) and (averaged) locked form burial are studied (Figure 3). There is an interaction of equivalent helices in the trimer toward the C terminus, which is only present for some structures. Other than this, major regions of interaction differences between 7krq and the closed form (555–670) and (830–860) have, by contrast, similar monomer burial in 7krq (neutral pH, D614G mutation, pocket closed) and the locked form (neutral pH, linoleic acid bound). These two regions contact between monomers in the trimer, and it has been suggested that a salt-bridge lost between D614 and K854 (of neighboring monomers) in variants carrying the D614G mutation effectively breaks a latch and leads to a greater fraction of open (RBD up) forms, thereby enhancing interaction with the receptor (Yurkovetskiy et al., 2020). Another interpretation of the effect of the D614G mutation is that it stabilizes the S protein trimer against dissociation (Zhang et al., 2021a), which is consistent with the hypothesis that once a destabilizing burial of D614 is lost, a locked form is more accessible at neutral pH (Lobo and Warwicker, 2021).

A further region of interest is (360–470), where there is most differentiation between the D614G 7krq structure and the locked/pocket occupied form (Figure 3). This segment is of interest for several reasons. Within it lie parts of the linoleic acid pocket, including the gating mechanism (Toelzer et al., 2020) (Figure 1). It also contains carboxylate groups of D405, D420, and E465 that are proposed (together with D614) to couple burial and pH-dependent stability (Lobo and Warwicker, 2021). Interestingly, in this region, the acid pH-locked structure 6xlu (Zhou et al., 2020) is more similar to the neutral pH-locked form than the D614G 7krq structure (Figure 3). If there were one or more buried and destabilized Asp/Glu side chains in this region, then such behavior would be expected.

Finally, the S protein of the Omicron variant (https://www.ecdc.europa.eu/en/covid-19/variants-concern) carries S371L, S373P, and S375F mutations in the sequence next to the RBD pocket gate. There are many further mutations in the Omicron S protein, which are being discussed in the context of various features, including the extent to which they mediate escape of an immune response primed by vaccine or prior infection (Greaney et al., 2021; Zahradník et al., 2021), and how immune escape mutations may be compensated to maintain ACE2 binding (Mannar et al., 2021) (Han et al., 2022). Interesting mutations in the Omicron spike protein, with respect to packing, are D614G and the S371L, S373P, and S375F cluster. The position equivalent to 373 in the RBD of coronavirus HKU9 is also a proline, and since this amino acid lies in a segment connecting two sides of the LA-binding pocket (in the SARS-CoV-2 spike), a proline substitution could alter main chain conformation and pocket gating. Indeed, the equivalent pocket region in HKU9 (5gyq) (Huang et al., 2016) looks to be closed, with a similar outcome anticipated for the RBD of the SARS-CoV-2 Omicron variant (Zahradník et al., 2021). If this pocket is closed in the Omicron variant spike, then linoleic acid would not bind and thus would not be a route to locking (compacting) the spike protein structure. It is intriguing that early reports of the Omicron spike structure indicate a capacity to form relatively compact structures in both closed (all RBD down) and open forms (Hong et al., 2022). Reports suggest that for the one RBD up form, this may be due, in part, to enhanced interactions between monomers (S375F–F486) (Zhou et al., 2021).

**FIGURE 4:**
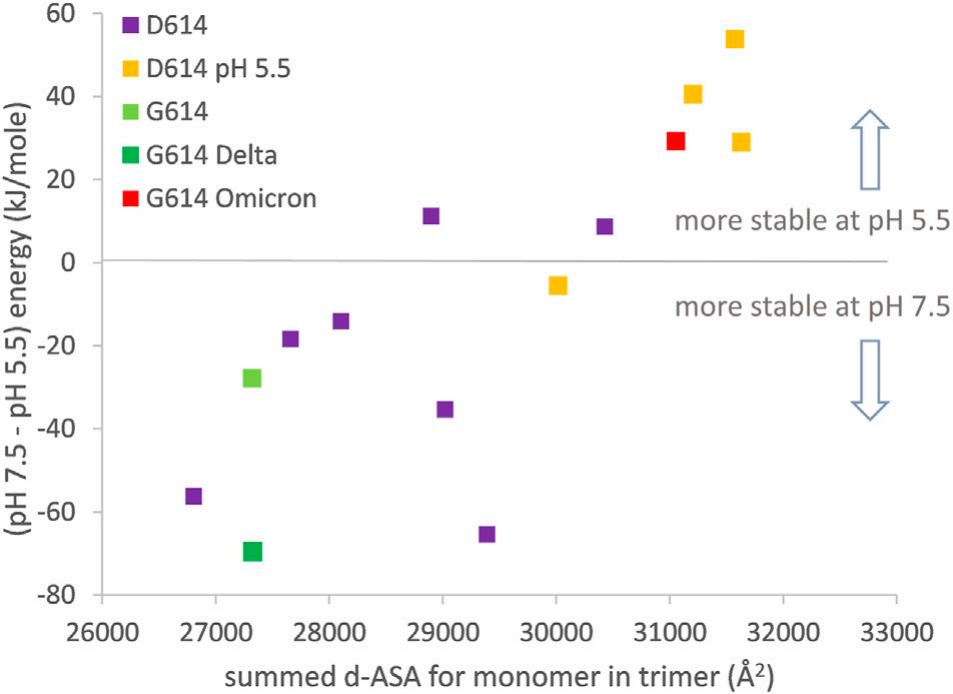
Compaction of the one RBD up trimer correlates to prediction of altered pH-dependent stability. Engineered (2P) one RBD up structures are (D614: 6xf6, 7knb, 7kne, 6xm3, 6xm4, 6xm0, 6vsb, 7byr, 6vyb, 7cn9, 6x2x; G614: 7bnn; G614-Delta 7w98; G614-Omicron 7tb4). d-ASA for each structure is summed over the three monomers in a trimer. Predicted pH dependence of trimer structural stability is calculated, following reported methods (Lobo and Warwicker, 2021), between pH 7.5 and pH 5.5.

The major thrust of pH dependence predictions for non-Omicron variants is that increased compaction of the spike trimer leads to partial dehydration of some Asp/Glu side chains and a disfavoring of a more compact form at neutral pH, unless balanced by linoleic acid binding to the RBD pocket (Lobo and Warwicker, 2021). In order to include the Omicron variant spike protein (which may not bind linoleic acid) in this picture, it is necessary to study the one RBD up form since this is the only currently available structure (January 2022) that has a complete set of three RBDs resolved (7tb4) (Zhou et al., 2021). Furthermore, to maintain equivalence between the engineered status of the spike protein ectodomain, analysis is restricted to the 2P (K986P, V987P) stabilized form. In keeping with the previous work (Lobo and Warwicker, 2021), increased trimer compaction correlates with a relative lowering of stability at pH 7.5 compared with pH 5.5 (Figure 4). The Omicron spike protein is predicted to be as compact as one RBD up trimers that have been stabilized by lowering the pH of structure solution to 5.5, although the Omicron spike was not solved at acidic pH. Presumably this increased compaction arises from interactions (including S375F–F486) that are absent in other SARS-CoV-2 variant spike proteins and that balance the predicted electrostatic destabilization at neutral pH. Since relatively compact trimers have been reported for both all RBD down and RBD up Omicron spike protein structures at neutral pH (Hong et al., 2022), likely in the absence of RBD pocket factor binding, it is reasonable to suggest that they may be better protected against S1 shedding than spike trimer of other SARS-CoV-2 variants. Furthermore, although a predicted pH dependence of stability remains for the Omicron variant spike, with an overall shift in the balance of trimer stabilizing interactions, it is unclear how this will translate to the prevalence of different spike conformational forms in different pH environments. One possibility is that with relatively compact closed and open (at least one RBD up) forms, with similar electrostatic destabilization (due to partial Asp/Glu burial), transition between the forms would be facilitated.

## Concluding Remarks

The roles of factors that contribute to establishing pH sensing in biology have been discussed, including pH variation within and outside of cells, separating groups that are genuinely pH sensing (i.e., with pH titration that can be modulated) and those that couple to pH sensing (often through interactions at an interface), the importance of focusing on all groups that could titrate in a physiological pH range, and the roles that homology and omics data can play in large-scale screening for pH sensors.

In the example chosen, SARS-CoV-2 spike protein, reference is made to predictions of pH dependence with a large set of S protein trimer structures, classified into different conformational forms (Lobo and Warwicker, 2021). Although this area is currently busy with data collection, many mechanisms remain unknown, including the pH dependence of S trimer conformation and how that might relate to function. Several themes are apparent in the S protein work, including the use of a large number of input structures to capture variation, close examination of ionization site burial, and how that changes with structural classes, and proposing that Asp/Glu side chains could be responsible for observed pH-dependent effects. This proposal is yet to be tested, indeed an alternate hypothesis is that histidines H49 and H519 could mediate pH dependence (Qu et al., 2021), but these give small predicted pH dependence (Lobo and Warwicker, 2021) and are located in regions without large interface changes between structural forms (Figure 3). One challenge facing analyses of pH dependence is how to construct experiments that continue to test models and predictions as the systems investigated become larger and direct biophysical readouts more complex to measure.
